# Open questions: A rose is a rose is a rose - or not?

**DOI:** 10.1186/1741-7007-12-2

**Published:** 2014-01-31

**Authors:** Ronald N Germain

**Affiliations:** 1 National Institute of Allergy and Infectious Diseases, National Institutes of Health, 9000 Rockville Pike, Bldg 4 Rm 126A MSC 0421, Bethesda, MD 20892, USA

## 

The first to be afflicted were immunologists and hematologists, but now genomicists have succumbed to 'splitter’s disease'. 'Lumpers' have lost the fight, largely due to new technology, whether it is advanced flow cytometry methods such as mass spectrometry-based analysis of cells (CyTOF [1]) that can analyze more than 40 discrete parameters at one time, cell by cell, or methods for single cell transcriptomics [2-4]. For example, using CyTOF to analyze effector T lymphocytes, a cornucopia of apparently new functional cell types emerges - lymphocytes making these but not those cytokines in a mind-numbing array of combinatorial possibilities [5]. At the RNA level, each cell in what was thought to be a rather homogeneous population shows differential expression of transcripts across the genome [2-4]. Indeed, at a recent meeting, one presenter studying a gene whose allelic forms show strong quantitative expression variation at the RNA level (eQTL) in cell populations concluded that genetics don’t determine expression at the individual cell level. This was the interpretation of the data even though summing expression from each allele over many individual cells gave the same biased distribution of expression as seen using bulk populations, as of course must be true unless the technology is flawed.

What could this apparent lack of genetic regulation in single cells reflect? Only two major possibilities present themselves. The first is that each cell behaves differently, transcribing its allelic copies in an individualistic manner at relative levels that may differ substantially from the overall proportions seen at the population level. Somehow, however, the entire population adjusts its mix of these patterns in a communal fashion to give the overall bias in expression that is seen in bulk analyses. There are precedents for such population control of behavior that is stochastic at the individual cell level - Arthur Lander previously commented on this issue when discussing how hematopoietic stem cells maintain their numbers [6]. Rather than the stereotypic view that stem cell division is invariably asymmetric, giving rise to one daughter cell that differentiates and one that retains self-renewal capacity, he and others [7] propose that any given division of a stem cell may yield not only the classical distinct fates just mentioned but also either two differentiating daughter cells or two self-renewing cells; feedback monitoring of the results of such events ensures that these decisions are biased over time in the total stem cell population to yield a balance of differentiating and self-renewing cell progeny.

Such community-based feedback control is unlikely, however, to be the explanation for the genomicist’s data on eQTL and transcript abundance at the single cell level, given that the quantitative trait is encoded directly in the DNA. An alternative, much more likely explanation is asynchronous bursts of RNA generation from each allele [8,9]. As a consequence, any given cell at any given moment could be at the nadir of transcript abundance for the generally better expressed allele and near the apex of transcript accumulation of the generally less well expressed copy. At that instant, a snapshot of the cell will show a transcript ratio that does not reflect the time integral of expression that controls the protein level, which is likely to be less spikey. It is precisely the use of snapshot analysis rather than measurement of dynamic behavior that obscures the possibility (likelihood?) that, over time, the cell will have more transcripts and more protein representing the allele revealed at the population level to be more highly expressed. Evidence for this view of things comes from analysis of protein expression by immune cells and studies on the fate of hematopoietic stem cells (HSCs) with a range of surface marker expression. There is always some heterogeneity of protein expression, even amongst cells of the most stringently defined subpopulation. For some lymphocyte proteins, sorting the cells at the extremes of the distribution and then maintaining them in a viable state without cell division leads to recovery of the original distribution of protein expression seen for the entire starting population (JN Mandl and RN Germain, unpublished observations). This makes it clear that each cell does not occupy a narrow expression bin, with the overall population distribution arising from a sum of cell behaviors constrained to these small bins, but rather that each cell moves over time through the entire range of expression seen in the population, occupying a different position in this distribution at any moment. Likewise, for HSCs, sorting for specific cell surface markers produces subsets of cells biased in the tendency to develop along the myeloid or erythroid lineages under the influence of specific cytokines, but regrowth of the sorted cells in HSC-maintaining conditions results in regeneration of the original distribution of markers and cells in the myeloid- versus erythroid-biased microstates [10].

**Figure 1.**
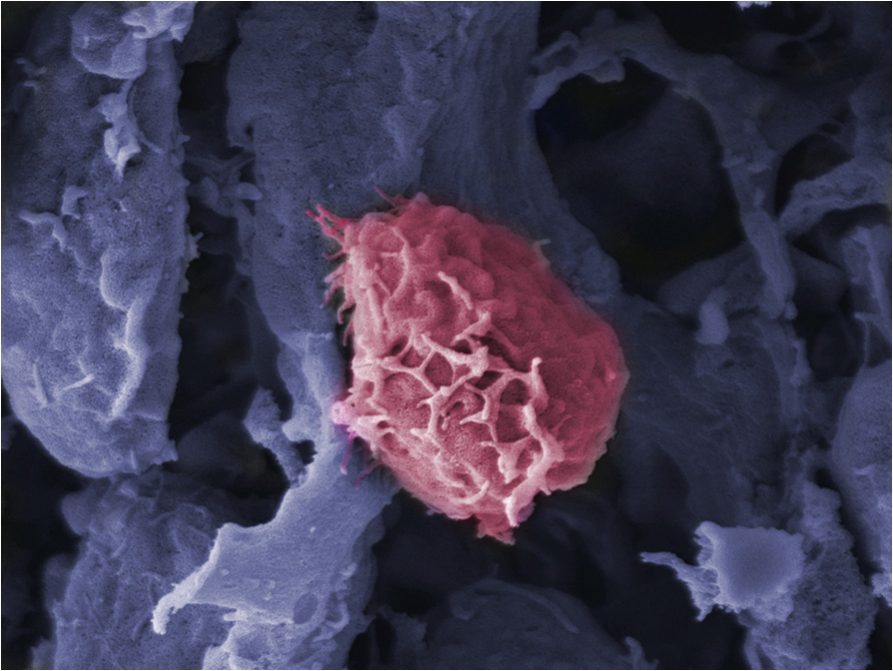
**Migrating T cell.** Scanning electron micrograph of a T cell (pink) crawling along stromal cells (blue) forming specialized conduits in the lymph node.

What does all this mean biologically? Interestingly, the snapshot view has some value in thinking about cell behavior. For example, T lymphocytes are activated in lymph nodes when they encounter dendritic cells displaying their specific antigen. A migrating T lymphocyte (Figure 1) searching for the right antigen-bearing cell will vary over time in its expression of key molecular components (integrins, kinases, and so on) involved in its ability to bind to the dendritic cell or to transmit a signal through the antigen receptor. At the moment it finds the relevant antigen-displaying dendritic cell, the T cell might be at the low end of the spectrum of expression of one or more of these key components and unable to mount an effective response (think of a sleep-derived student doing poorly on a difficult test). The same T cell some hours later meeting the same dendritic cell, but with high expression of the same crucial components, would 'ace the test’. Considered in this light, the instantaneous state of a cell as revealed by CyTOF or single cell transcriptomic studies could be relevant to how that cell functions biologically. However, we shouldn’t draw overly simplistic conclusions from such considerations - data from several labs indicate that cells expend energy trying to co-ordinate such fluctuations in expression among components in a pathway so as to limit excursions of cell state to the extremes of hyper- and hypo-responsiveness [11,12], so one needs to take care to examine the overall functionality of such networks and not just the expression of an individual member in evaluating momentary cell behavior.

Given the evidence that cell state changes over time, it seems clear that if the time constant of such fluctuation is short and the length of exposure to a stimulus substantially longer, each cell in a population might show 'average’ behavior rather than the highly individualistic functionality described above [10-13]. And even if this were not the case, cells of the same differentiation type that are not in temporal synchrony for their state variation would show in an ensemble manner the average behavior represented by the distribution of signaling capacities in the population - in many cases, this would represent the relevant biology, not the instantaneous single cell variation in response [13]. Whether the personalized, snapshot state or this population average state is of predominant importance *in vivo* might vary with the size of the relevant cell subset. For nearly unique immune cells with clonal receptors, it would be difficult to average out the response over a population and the instantaneous state could predominate at the moment of cell triggering by antigen, whereas for cells lacking such clonality, the distributed population behavior could be more relevant.

Even for immune cells, however, one needs to be cautious about inferring too much from snapshot data, such as flow studies at a fixed time after stimulation. There will be temporal variation in the onset and peak of transcription and translation of products of distinct cytokine loci, for example, and thus, a population of effector cells will appear much more heterogeneous in their behavior when analyzed at one point than if each cell was followed over time to see the entire set of responses made to the stimulus. This perspective emphasizes the critical value of dynamic analysis of molecular expression in single cells for understanding cell fate decisions [14,15].

We have entered an era in which our technologies enable us to collect increasingly fine-grained data about cells and their states and the resulting insight into cellular heterogeneity poses important issues for biologists. With this new knowledge comes a responsibility to avoid being so enamored of the methodology that we lose track of the meaning of the data in the context of an integrated biological system. As I have noted here, pictures of cells frozen in time can be useful, but also misleading with respect to each one’s ultimate behavior as well as that of the population to which they belong. We need to tell the roses from the daffodils, but also be careful not to call two roses a rose and a lily.
